# Mortality and its predictors among patients with Guillain–Barré syndrome in the intensive care unit of a low-income country, Ethiopia: a multicenter retrospective cohort study

**DOI:** 10.3389/fneur.2024.1484661

**Published:** 2024-10-30

**Authors:** Habtu Tsehayu Bayu, Atalay Eshetie Demilie, Misganew Terefe Molla, Fantahun Tarekegn Kumie, Amanuel Sisay Endeshaw

**Affiliations:** Department of Anesthesia, College of Medicine and Health Science, Bahir Dar University, Bahir Dar, Ethiopia

**Keywords:** Mortality, Predictors, GBS, Guillain-Barré syndrome, ICU, Ethiopia

## Abstract

**Background:**

Guillain–Barré syndrome (GBS) is a rare autoimmune disease that affects the peripheral nervous system. It is characterized by the destruction of nerves involved in movement. This condition can lead to transient pain, changes in temperature and touch sensations, muscle weakness, loss of sensation in the legs and/or arms, and difficulty swallowing or breathing. Published data on the outcomes of critical care for patients with GBS are extremely scarce in Africa, particularly Ethiopia. Therefore, this study aimed to assess mortality and its predictors among patients with GBS in the intensive care unit (ICU) of specialized hospitals in Ethiopia, a low-income country.

**Materials and methods:**

This retrospective cohort study was conducted at the Tibebe Ghion Specialized Hospital and the Felege Hiwot Comprehensive Specialized Hospital in Bahir Dar, Ethiopia, from 1 January 2019 to 30 December 2023. Data were collected in the medical record rooms. Cox regression analysis was performed to identify the predictors of mortality among GBS patients in the ICU. The crude and adjusted hazard ratios (AHRs) and 95% confidence intervals (CIs) were calculated using bivariable and multivariable Cox regression models. A *p*-value of <0.05 was considered statistically significant.

**Results:**

Of 124 GBS patients admitted to the ICU, 120 were included in the final analysis. During the follow-up, there were 23 (19.17%) deaths. The overall incidence rate of death was 1.96 (95% CI: 1.30, 2.95) per 100 person-days of observation. Traditional medicine (AHR = 3.11, 95%: 1.12, 16.70), COVID-19 infection (AHR = 5.44, 95% CI: 1.45, 73.33), pre-ICU cardiac arrest (AHR = 6.44, 95% CI: 2.04, 84.50), and ICU readmission (AHR = 4.24, 95% CI: 1.03, 69.84) were identified as the independent predictors of mortality.

**Conclusion:**

The mortality rate among GBS patients admitted to the ICU was high. Traditional medicine, COVID-19 infection, pre-ICU cardiac arrest, and readmission to the ICU were the significant predictors of mortality. Conducting large-scale studies with a prospective design in the future would yield more robust evidence.

## Introduction

Guillain–Barré syndrome (GBS) is a rare autoimmune disease that affects the peripheral nervous system. It is characterized by the destruction of nerves involved in movement. This condition can lead to transient pain, changes in temperature and touch sensations, muscle weakness, loss of sensation in the legs and/or arms, and difficulty swallowing or breathing (1, 2). GBS is usually caused by a preceding bacterial or viral infection, such as *Campylobacter jejuni*, *Cytomegalovirus*, Epstein–Barr virus, or *Mycoplasma pneumoniae* (3).

According to the 2019 Global Burden of Disease report, there were 150,095 cases of GBS worldwide, with a point prevalence of 1.5 cases per 100,000 people (4). Moreover, the worldwide incidence rate of GBS is approximately 1–2 cases per 100,000 person-years (5, 6). The global distribution of GBS varies by region and income level, with a higher number of cases reported in high-income countries (HICs) in North America and East Asia compared to other regions (4). However, mortality rates among GBS patients are higher in low-and middle-income countries (LMICs) compared to HICs. This disparity is attributed to delayed diagnosis and more severe disease presentations due to insufficient diagnostic and healthcare facilities in LMICs (7).

Admission to an intensive care unit (ICU) for mechanical ventilation and other organ support is the primary treatment strategy for GBS patients, particularly those with severe disease presentations (8, 9). Studies have shown that more than a quarter of GBS patients require ICU admission (10). The primary indications for ICU admission among GBS patients include respiratory failure, dysautonomia, tracheostomy for prolonged ventilation, significant electrolyte imbalances, and multi-organ failure (11, 12).

The literature from developed countries reveals that ICU mortality rates for GBS patients range from 3.9 to 11% (13, 14), while over half of GBS patients admitted to ICUs in developing countries do not survive (15). In African studies, ICU mortality rates among GBS patients ranged from 11.4 to 15.38% (16, 17). Previous studies conducted in tertiary hospitals in Ethiopia have reported that ICU mortality rates among GBS patients ranged between 16.4 and 25.9% (18, 19). Several factors have been identified as predictors of mortality among patients with GBS in critical care settings, including advanced age, pre-existing chronic respiratory disease, the need for mechanical ventilation, pre-ICU cardiorespiratory arrest, immunocompromised status, prolonged ICU stay, COVID-19 infection, and acute motor axonal neuropathy (13, 20–22).

Published data on the outcomes of critical care for patients with GBS are extremely scarce in Africa, including Ethiopia. Most studies on the intensive care management of GBS patients originate from high-income countries and may not be applicable to LMICs due to differences in access to and quality of care for critically ill patients with GBS. Therefore, this study aimed to assess mortality and its predictors among patients with GBS in the ICU of specialized hospitals in a low-income country, Ethiopia.

## Materials and methods

### Study design, period, and setting

This retrospective cohort study was conducted at the Tibebe Ghion Specialized Hospital and the Felege Hiwot Comprehensive Specialized Hospital in Bahir Dar, Ethiopia, from 1 January 2019 to 30 December 2023. Both specialty hospitals are located in Bahir Dar, the capital city of the Amhara Region, in northwest Ethiopia, 580 km from Addis Ababa, the capital city of Ethiopia.

The Tibebe Ghion Specialized Hospital was established in December 2018 and provides clinical and academic services in affiliation with Bahir Dar University. This hospital has a total of 500 beds, including 18 ICU beds, and 1,000 healthcare providers. The ICU staff includes 1 pulmonologist, 1 medical neurologist, 23 anesthetists, and 35 critical care nurses. The Felege Hiwot Comprehensive Specialized Hospital was established in 1971 and serves more than seven million people in the surrounding area. The facility contains 490 beds, including 12 ICU beds. The hospital has a total of 900 healthcare providers. Among them, the number of healthcare providers in the ICU includes 30 critical care nurses, 20 anesthesia providers, and 3 senior specialists.

We used the Strengthening the Reporting of Observational Studies in Epidemiology (STROBE) checklist for reporting the results of this study (23).

### Participants

All patients diagnosed with GBS admitted to the ICU at the Tibebe Ghion Specialized Hospital and the Felege Hiwot Comprehensive Specialized Hospital from 1 January 2019 to 30 December 2023 were eligible for inclusion. Patients with unknown outcome status and lost medical records were excluded from the study.

### Data collection method and quality control

Data collection was conducted in the medical record rooms of the Tibebe Ghion Specialized Hospital and the Felege Hiwot Comprehensive Specialized Hospital. To ensure data accuracy, an initial assessment of the medical records for eligible patients was performed using the ICU registration books of both hospitals before the day of data collection. Two experienced BSc anesthetists, under the supervision of an MSc anesthetist, collected the data required for this study. Before the data collection, data collectors and the supervisor provided a 1-day simulation-based training that included both the process and ethics of data collection. The data were collected from 13 January 2024 to 17 April 2024. The data collected were kept confidential by using codes, and access was restricted.

## Variables of the study

### Outcome variables

The study’s primary outcome was the time to death following admission to the ICU. Patients with GBS who died while in the ICU were considered to have experienced the event, while those who did not were labeled as censored.

### Explanatory variables

Explanatory variables were divided into three categories: (1) demographic variables: age (in years), sex, and residence; (2) clinical variables: duration of illness, traditional medicine, motor weakness, area of extremity weakness onset, comorbidity, COVID-19 infection, pre-ICU cardiac arrest, mechanical ventilation, complications in the ICU, and readmission to the ICU; and (3) biochemical variables: abnormal glycemic level, abnormal potassium level, abnormal sodium level, abnormal liver function test, and creatinine level.

### Data management and analysis

The data were coded and entered into Epi-Data version 4.6, then exported to STATA version 17 for analysis. Cross-tabulations and summary statistics were performed to describe the study population and relevant factors, ensuring data completeness and accuracy. Descriptive results were summarized using tables and graphs.

The Kaplan–Meier failure curves and log-rank tests were used to compare survival differences among categorical variables. Cox regression analysis was used to identify the predictors of mortality among GBS patients in the ICU. The proportional hazards assumption was checked using a global Schoenfeld residual test (*p* = 0.2711). Bivariable and multivariable Cox regression models were used to compute crude and adjusted hazard ratios (AHRs) and 95% confidence intervals (CIs). A *p*-value of <0.05 was considered statistically significant.

### Ethical approval and consent to participate

This study was approved by the institutional review board (IRB) of the College of Medicine and Health Science, Bahir Dar University (Reference number: 856/2023), and Felege Hiwot Comprehensive Specialized Hospital. The institutional review board (IRB) of the College of Medicine and Health Science, Bahir Dar University, and Felege Hiwot Comprehensive Specialized Hospital waived the need for written informed consent from all study subjects, as the research utilized secondary data. All methods were carried out in accordance with applicable guidelines and regulations.

## Results

### Demographic characteristics of GBS patients admitted to the ICU

During the study period, 124 GBS patients admitted to the ICU were included. Four patients were excluded due to incomplete data, leaving a total of 120 patients in the final analysis.

The majority of GBS patients admitted to the ICU were under 18 years, accounting for 56 (46.66%) of the total admissions. The majority of GBS patients admitted to intensive care units were male individuals, comprising 71 (59.17%) of the total hospitalized patients. Approximately 75 (62.50%) GBS patients lived in rural areas (Table 1).

**Table 1:** Demographic characteristics of GBS patients admitted to the ICU from 1 January 2019 to 30 December 2023, Bahir Dar, Ethiopia.

Variables	Category	Frequency	Percentage
Age (in years)	<18	56	46.66
	19–65	55	45.83
	>65	9	7.51
Sex	Male	71	59.17
	Female	49	40.83
Residence	Rural	75	62.50
	Urban	45	37.50

### Clinical and biochemical profiles of patients with GBS admitted to the ICU

Of all GBS patients admitted to the ICU, 62 (51.7%) had a duration of illness of less than 3 days and 18 (15.00%) had used traditional medicine. Approximately all (99.17%) of the study participants experienced motor weakness. Among those with extremity weakness, approximately two-thirds (66.39%) reported that motor weakness began in the proximal part of the extremity. Of all study subjects, 41 (34.17%) had comorbidities and 8 (6.67%) had COVID-19 infection. Regarding the biochemical profile of GBS patients admitted to the ICU, 74 (61.7%) had dysglycemia. Electrolyte abnormalities, such as potassium and sodium imbalances, were observed in 39.2 and 63.3% of GBS patients, respectively. Approximately three-fourths (71.67%) of the study participants had abnormal liver function tests, and 55 (45.83%) had creatinine levels greater than 1 mg/dL. In addition, 15 (12.5%) patients were readmitted to the ICU, and 10 (8.33%) patients experienced pre-ICU cardiac arrest (Table 2).

**Table 2:** Clinical and biochemical characteristics of GBS patients admitted to the ICU from 1 January 2019 to 30 December 2023, Bahir Dar, Ethiopia.

Variable	Category	Frequency	Percentage
Duration of illness	<3 days	62	51.67
	3 days–21 days	47	39.17
	>21 days	11	9.16
Traditional medicine	Yes	18	15.00
	No	102	85.00
Motor weakness	Yes	119	99.17
	No	1	0.83
Area where extremity weakness started (*n* = 66)	Proximal	43	65.15
	Distal	23	34.85
Comorbidity	Yes	41	34.17
	No	79	65.83
COVID-19 infection	Yes	8	6.67
	No	112	93.33
Abnormal glycemic level	Yes	74	61.67
	No	46	38.33
Abnormal potassium level	Yes	47	39.17
	No	73	60.83
Abnormal sodium level	Yes	76	63.33
	No	44	36.67
Abnormal liver function test	Yes	86	71.67
	No	34	28.33
Creatinine level	≤1	65	54.17
	>1	55	45.83
Pre-ICU cardiac arrest	Yes	10	8.33
	No	110	91.67
Complications in the ICU	Yes	109	90.83
	No	11	9.17
Readmission to the ICU	Yes	16	13.33
	No	104	86.67

### Management of GBS patients in the ICU

**Figure 1:**
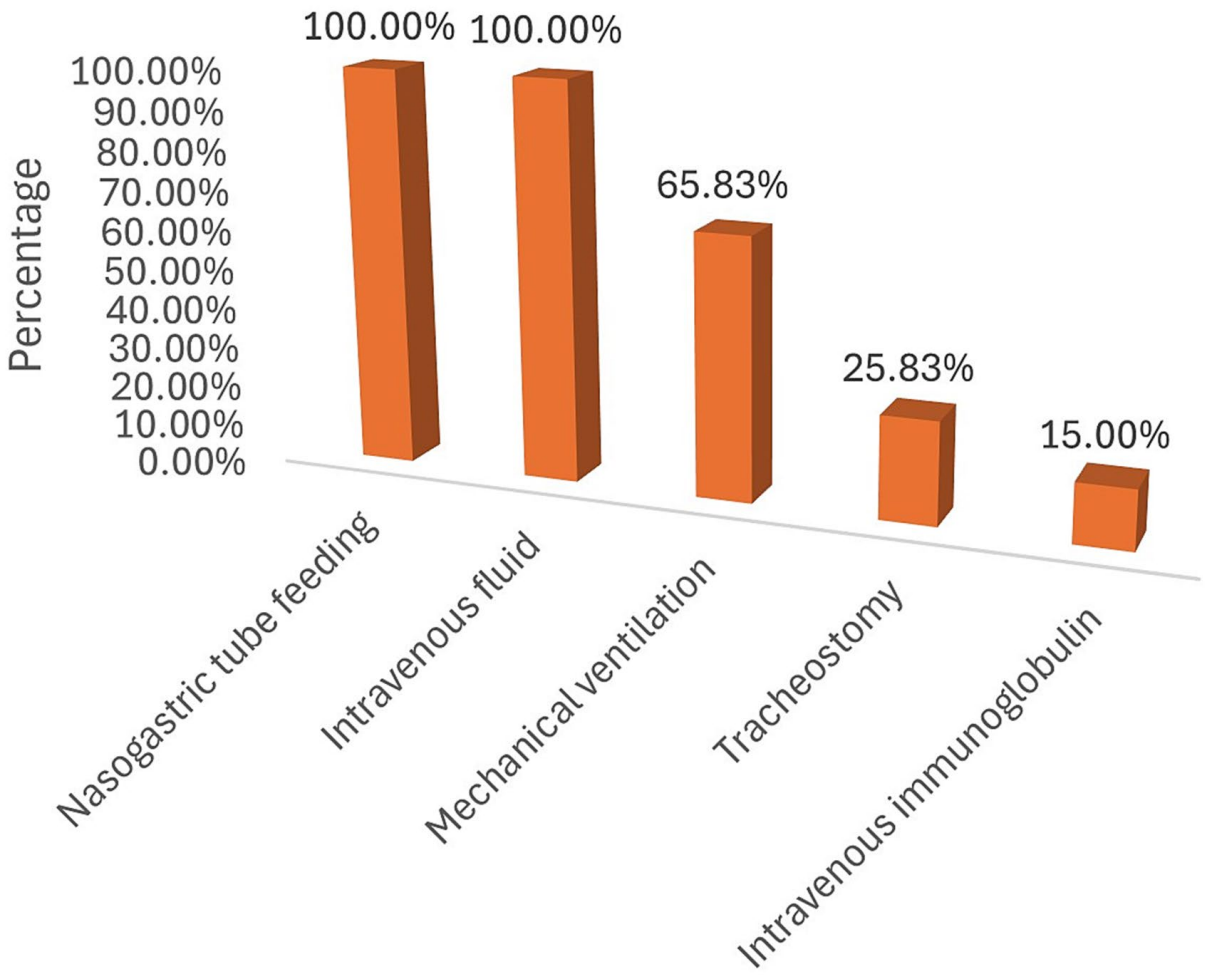
Treatment provided for GBS in the ICU.

**Figure 2:**
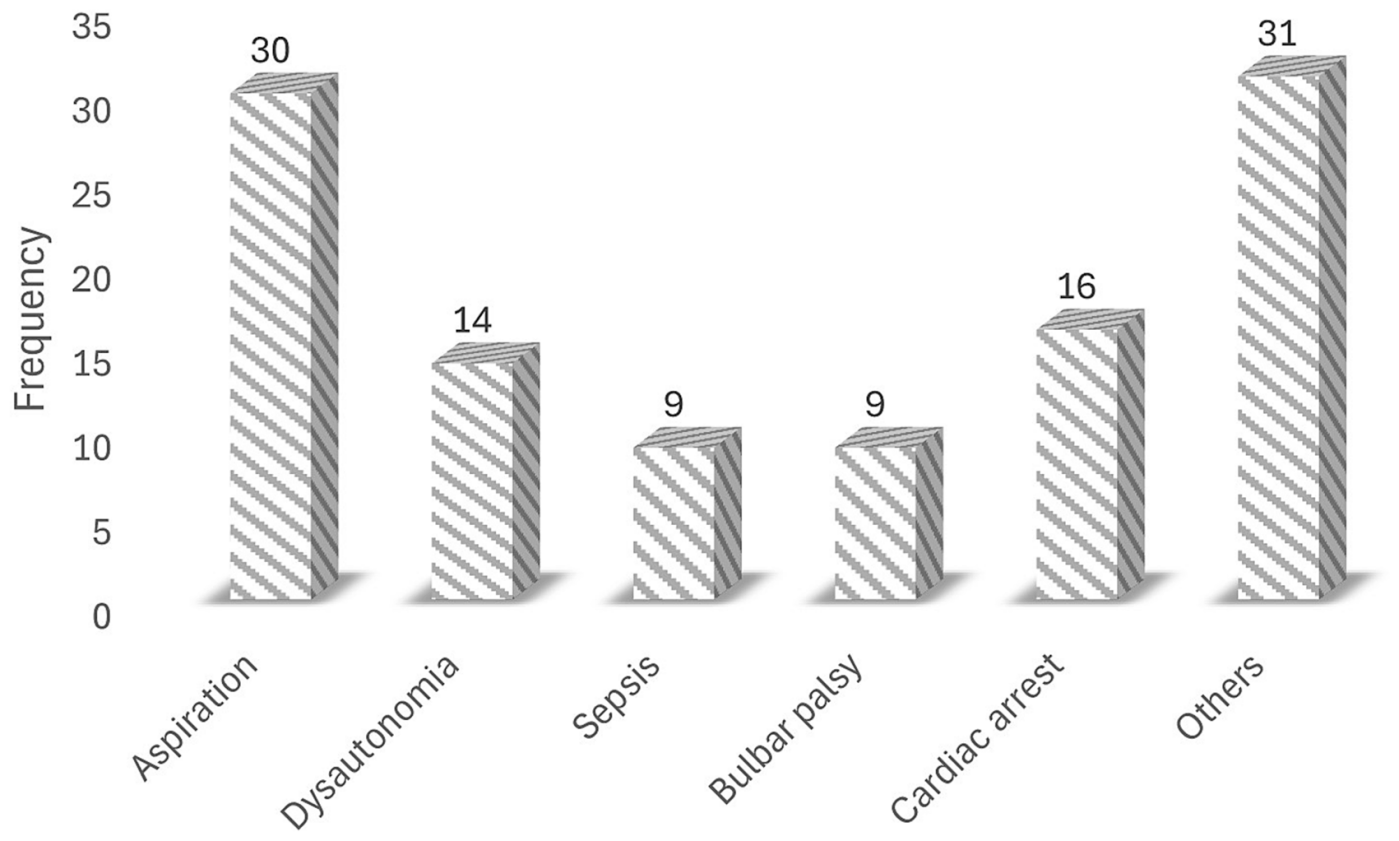
Common complications in the ICU among patients with GBS (other = anemia, hypotension, and malnutrition).

Of the 120 GBS patients admitted to the ICU, 79 (65.83%) required mechanical ventilation, 31 (25.83%) had a tracheostomy for prolonged ventilation, and 18 (15.00%) received intravenous immunoglobulin. During their stay in the ICU, 109 patients experienced at least one complication (Figure 1). The most frequently recorded complication was aspiration (34/109), followed by cardiac arrest (16/109) (Figure 2).

### Survival analysis

A total of 120 participants were tracked for a total of 1,171 person-days. Participants were observed for a median of 6 days, ranging from a minimum of 1 day to a maximum of 60 days. During the follow-up period, there were 23 (19.17%) deaths, while the remaining 97 (79.93%) patients were censored. Among them, 70 (58.33%) were discharged home, 22 (18.33%) left against medical advice, and 5 (4.217%) were referred to other hospitals.

**Figure 3:**
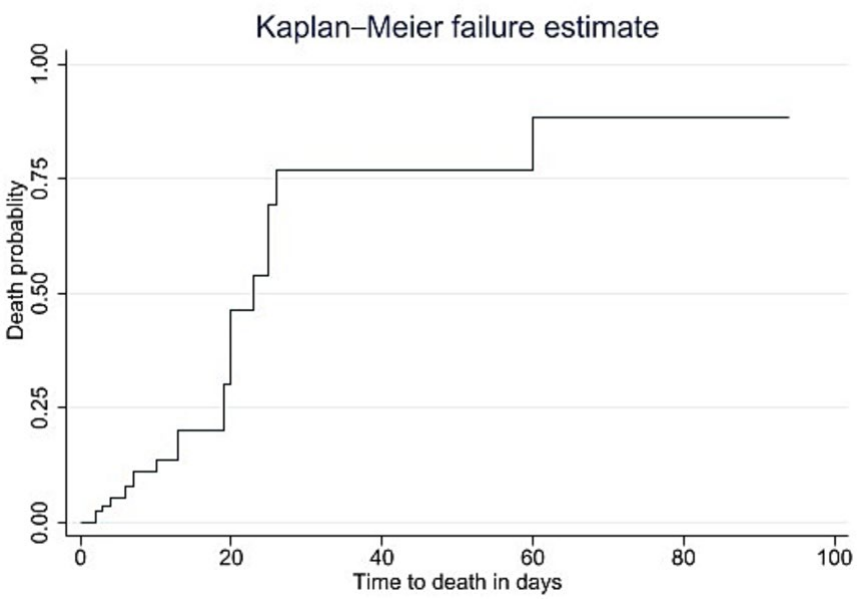
Kaplan–Meier failure curves of mortality among patients with GBS admitted to the ICU from January 2019 to December 2023, Bahir Dar, Ethiopia.

The overall incidence rate of death was found to be 1.96 deaths per 100 person-days of observation (95% CI: 1.30, 2.95). The median time to death for GBS patients in the ICU was 23 days (interquartile range (IQR) 20, 26). The cumulative probability of death for GBS patients admitted to the ICU was 13.04% on the first day of admission and 95.65% at 60 days post-admission. Regarding the time to death, the Kaplan–Meier failure curves indicated that the mortality risk for patients with GBS admitted to ICU increased with a longer length of stay in the ICU (Figure 3).

### Predictors of mortality among GBS patients admitted to the ICU

We used a log-rank test to compare survival differences among categorical variables. Among all variables considered, there were statistically significant differences in survival related to traditional medicine, comorbidities, COVID-19 infection, abnormal potassium levels, pre-ICU cardiac arrest, and readmission to the ICU.

After performing multivariable Cox regression analysis, covariates such as traditional medicine, COVID-19 infection, pre-ICU cardiac arrest, and ICU readmission were identified as independent predictors of mortality among critically ill patients with GBS. The risk of mortality among GBS patients who used traditional medicine before ICU admission was three times higher (AHR = 3.11, 95%: 1.12, 16.70) than that of their counterparts. GBS patients who had COVID-19 infection faced a 5.44 times (AHR = 5.44, 95% CI: 1.45, 73.33) higher risk of death in the ICU compared to patients without COVID-19 infection. Patients with pre-ICU cardiac arrest had a 6.44 times higher risk of mortality (AHR = 6.44, 95% CI: 2.04, 84.50) compared to those without a history of cardiac arrest. The risk of death for GBS patients who were readmitted to the ICU was 4.24 times higher (AHR = 4.24, 95% CI: 1.03, 69.84) than for those admitted for the first time (Table 3).

**Table 3:** Predictors of mortality among GBS patients admitted to the ICU from 1 January 2019 to 30 December 2023, Bahir Dar, Ethiopia.

Variables	Category	Died	Censored	CHR (95%CI)	AHR (95%CI)
Traditional medicine	Yes	8	10	4.37 (1.8, 11.6)	3.11 (1.12, 16.70)*
	No	15	87	1, ref	1, ref
Comorbidity	Yes	19	60	0.27 (0.08, 2, 06)	4.07 (0.27, 60.08)
	No	4	37	1, ref	1, ref
COVID-19 infection	Yes	5	3	16.53 (4.72, 57.58)	5.44 (1.45, 73.33)*
	No	18	94	1, ref	1, ref
Abnormal potassium level	Yes	22	25	16.54 (2.17, 125.80)	1.70 (0.04, 31.85)
	No	1	72	1, ref	1, ref
Pre-ICU cardiac arrest	Yes	9	1	6.91 (2.82, 16.84)	6.44 (2.04, 84.50)**
	No	14	96	1, ref	1, ref
Readmission to the ICU	Yes	6	10	3.88 (1.44, 10.25)	4.24 (1.03, 69.84)*
	No	17	87	1, ref	1, ref

CHR, Crude hazard ratio; CI, confidence interval; AHR, adjusted hazard ratio. *P-value < 0.05 and **P-value < 0.001.

## Discussion

This retrospective cohort study primarily assessed mortality and its predictors among GBS patients admitted to the ICU at specialized hospitals in Ethiopia, which is a low-income country. These findings indicated that mortality in the ICU among patients with GBS is high, with traditional medicine, COVID-19 infection, pre-ICU cardiac arrest, and readmission to the ICU being significant predictors of mortality. This study’s clinical importance lies in providing baseline information for care providers and health service managers regarding the outcomes of critically ill patients with rare neurologic diseases (such as GBS) in resource-constrained settings, which can guide efforts to improve clinical outcomes.

In this study, the overall incidence of mortality among GBS patients admitted to the ICU at two specialized hospitals in Bahir Dar, Ethiopia, was found to be 19.17%, with a rate of 1.96 deaths per 100 person-days of observation. This finding is consistent with the results of studies conducted in Serbia (24), Ethiopia (18), and India (25), which reported mortality rates of 16.2, 16.4, and 21%, respectively. However, the mortality rate in our study is higher than that reported in studies conducted in Canada (11) and Asian countries (26). The discrepancy might be due to differences in access and quality of intensive care in HIC, which contribute to a lower mortality rate.

GBS patients who used traditional medicine were found to have a significantly increased risk of death in the ICU. This finding aligns with a study conducted in South Africa (17). One possible explanation for this association is that traditional medicines might have induced liver injury, which increased the likelihood of death in the ICU (27, 28). Apart from the clinical association, patients with GBS who used traditional medicine were found to have a higher risk of delaying treatment and experiencing severe disease presentations, which negatively affects outcomes after critical illness. However, there is contrary evidence from China indicating that traditional medicine is associated with a lower risk of death (29). The integration of traditional medicine into modern clinical practice in China, approached in a scientific manner, may explain this discrepancy. Our study found that GBS patients who were infected with COVID-19 had a higher risk of death in the ICU. This finding is consistent with an Italian study (30). The best explanation for this might be that patients with COVID-19 have a higher chance of respiratory complications, such as respiratory failure and pneumonia, which may increase the risk of death (31, 32).

According to the findings of studies conducted in Australia, New Zealand, and the United States of America (13, 33), pre-ICU cardiac arrest was identified as an independent predictor of mortality in the ICU among GBS patients. The main reason may be that patients who experience cardiac arrest are highly susceptible to post-cardiac arrest complications, such as ventricular fibrillation, ventricular tachycardia, and pulseless electrical activity, which increase the likelihood of mortality (34). This study showed that readmission to the ICU among GBS patients is associated with an increased risk of mortality. This finding aligns with a study conducted by Damian et al. (34). A possible reason for this might be that readmitted patients have more severe disease patterns, which increase the likelihood of sepsis and organ failure, negatively impacting patient survival (35, 36).

A limitation of this study is that, due to its retrospective design, certain vital characteristics of the study participants, such as nerve conduction profiles and MRC scores, were unavailable. We conducted the study using a small sample size, which may affect its generalizability. Furthermore, significant predictors of ICU outcomes, such as the APACHE II score, albumin level, and SOFA score, were not included in the analysis due to the study design.

## Conclusion

This retrospective cohort study revealed a high mortality rate among GBS patients admitted to the ICU of two specialized hospitals in Ethiopia. Traditional medicine, COVID-19 infection, pre-ICU cardiac arrest, and readmission to the ICU were identified as significant predictors of mortality in the ICU among patients with GBS.

Based on the findings of the study, we recommend that clinicians working in critical care implement target-specific interventions for patients with GBS who present with the identified predictors. Large-scale studies with a prospective design would provide more robust evidence in this regard among GBS patients admitted to the ICU.

## Data Availability

The raw data supporting the conclusions of this article will be made available by the authors, without undue reservation.
